# The Treatment of Scabies by Chlorine Gas

**Published:** 1918-01

**Authors:** 


***The Treatment of Scabies by Chlorine Gas.*** By G. Herbert Clark, Capt. R. A. M. C, and H. S. Raper, Temp. Capt. R.A.M.C. Abs. from the *Brit. Med. Jour.*, July 28, 1917.

C. and R. undertook experiments with the chlorine gas treatment, because an officer called their attention to the fact that after drill in anti-gas measures, during which a camp was repeatedly subjected to long exposures to gas, the entire camp was freed from scabies.

The authors have attempted the cure on 74 patients (commissioned and non-commissioned officers and men) with unquestionable cases of scabies.

The patients were exposed to a concentration of 1 or 2 parts chlorine to 1000 of air. The service gas helmet was used. The men were exposed for slightly varying periods of time and at various intervals. The number of exposures given to each man varied from 3 to 6. Each exposure lasted for 5 minutes.

Of the 60 cases of non-commissioned officers and men, only 25 o/o were pronounced definitely cured, although many cases showed much improvement. Of the 14 officers, 11 were definitely cured, and the rest much improved. The comparative failure with the men, the authors believe, was due to insufficient exposure and to the small gas concentration — not more than 1 to 1000.

The authors conclude that four exposures are generally enough to effect a cure, if they are given on the first, second, fifth, and sixth days.

They state, however, that a slight irritation is likely to result from exposures to the gas, felt especially at the scrotum and axillae. Washing soda added to the daily bath relieves this condition, and it passes off in the course of two weeks.

The gas cure may be due either to the direct toxic effect of the gas upon the acarus, or to the action of the hydrochloric acid liberated by the combination of the chlorine with the outer layer of the skin.

The authors cite a further attempt to check the spread of scabies by the chlorine treatment which proved entirely successful for a complete mounted section. The treatment was given in the sleeping quarters, and all clothing and bedding exposed at the same time. No further cases occurred in the unit for more than two months.

This method of treatment, the authors think, is at present easier to carry out, because of the gas equipments everywhere at hand, than the treatment by sulphur vapor or baths.

(*Note also the article on scabies abstracted in* The Medical Bulletin, *No. 1, p. 71.*)

